# Fifteen-year experience with pericardiectomy at a tertiary referral center

**DOI:** 10.1186/s13019-021-01561-4

**Published:** 2021-06-22

**Authors:** Zainab Faiza, Anjali Prakash, Niharika Namburi, Bailey Johnson, Lava Timsina, Lawrence S. Lee

**Affiliations:** Division of Cardiothoracic Surgery, Indiana University School of Medicine, Indiana University Health Methodist Hospital, 1801 N. Senate Blvd., Suite 3300, Indianapolis, IN 46202 USA

**Keywords:** Pericardiectomy, Constrictive pericarditis, Postoperative outcomes

## Abstract

**Purpose:**

Pericardiectomy has traditionally carried relatively high perioperative mortality and morbidity, with few published reports of intermediate- and long- term outcomes. We investigated our 15-year experience performing pericardiectomy at our institution.

**Methods:**

Retrospective study of all patients who underwent pericardiectomy at our institution between 2005 and 2019. Baseline demographics, intraoperative details, and postoperative outcomes including long-term survival were analyzed.

**Results:**

Sixty-three patients were included in the study. 66.7% of subjects underwent isolated pericardiectomy while 33.3% underwent pericardiectomy concomitantly with another cardiac surgical procedure. The most common indications for pericardiectomy were constrictive (79.4%) and hemorrhagic (9.5%) pericarditis. Preoperatively, 76.2% of patients were New York Heart Association class II and III, while postoperatively, 71.4% were class I and II. One-, three-, five-, and ten- year overall mortality was 9.5, 14.3, 20.6, and 25.4%, respectively. Overall pericarditis recurrence rate was 4.8%.

**Conclusion:**

Pericardiectomy carries relatively high overall mortality rates, which likely reflects underlying disease etiology and comorbidities. Patients with prior cardiac intervention, history of dialysis, and immunocompromised state are associated with worse outcomes.

## Introduction

Pericarditis can lead to scarring and adherence of the visceral pericardium to the epicardial surface [1]. Left untreated, this can lead to loss of pericardial compliance and subsequent diastolic heart failure. While most patients with pericarditis are managed medically, pericardiectomy, also referred to as pericardial stripping, is the definitive treatment for patients with unrelenting symptoms of heart failure [1–7]. Pericardiectomy is typically performed in patients with constrictive pericarditis (CP) where the extent of inflammation has led to fibrosis, scarring, and often calcification of pericardial tissue, thus impeding proper ventricular function [8, 9]. Pericarditis is diagnosed in up to 5% of patients with chest pain, with only a smaller subset progressing to chronic or CP. It is estimated that about 20% of patients diagnosed with CP ultimately undergo pericardiectomy [9–12]. In developed countries, the cause of pericarditis is unclear, although a history of viral infections, prior cardiac surgeries and mediastinal radiation are reported to be predisposing factors [2, 10, 13–15]. In fact, the incidence of CP in the 2 years following any cardiac surgical procedure is approximately 0.2–0.4% [4]. In developing countries, tuberculosis (TB), often associated with concomitant Human Immunodeficiency Virus (HIV) infection, is the leading cause of pericarditis and estimated to be the etiology in 22 to 91% of cases [9, 11, 13, 16, 17].

Patients with CP can have unremitting symptoms and recurrence despite medical management and multiple percutaneous attempts at treatment [4, 10, 16, 18, 19]. Surgery is often the only chance at definitive treatment in such cases. In the United States, 21% of patients admitted for CP ultimately undergo pericardiectomy [11]. Perioperative mortality following pericardiectomy has been relatively high, ranging from 2 to 15%, with variable long-term outcomes [1–3, 7–9, 11, 14, 20–23]. We sought to assess the risk factors and perioperative outcomes of patients undergoing pericardiectomy at our institution over a 15-year time interval.

## Patients and methods

This single-center retrospective study was approved by the Institutional Review Board (IRB) of Indiana University and conducted in accordance with the Declaration of Helsinki as well as all University guidelines and regulations. Informed consent by individual study patients was waived by the IRB. A prospective institutional database and Society of Thoracic Surgeons (STS) registry were queried to identify all patients who underwent pericardiectomy at our institution between 2005 and 2019. Patient demographics, intraoperative variables, and postoperative outcomes were extracted from the aforementioned data sources. Long-term survival was also assessed utilizing publicly available sources such as the Social Security Death Index and the Indiana State Office of Vital Statistics.

Primary outcomes analyzed included survival (at 1, 3, 5, and 10 years) and recurrence of pericarditis symptoms. Secondary outcomes analyzed included immediate postoperative complications, postoperative length of stay (LOS), and New York Heart Association (NYHA) functional classification.

### Statistical analysis

Given the study’s overall and subgroup sample size, and as evidenced from the Shapiro-Wilk W-test for normality, we performed descriptive analysis using median (Interquartile Range, IQR) for continuous variables. For categorical variables, frequency and percentages were reported. Bivariate analyses were done using Chi-square or Fisher’s exact tests for categorical variables and two-sample Wilcoxon rank-sum tests for continuous variables. Bivariate survival analysis was performed using log-rank test with Kaplan-Meier plots for equality of survivor functions between different types of pericardiectomy procedures. Multivariable survival analysis was done using Cox-Proportional Hazard model with backward stepwise method and with significance levels greater than 0.25 and less than 0.10 as the cutoffs, respectively, for removal from and addition to the model. We began our model with type of pericardiectomy and list of covariates (including preoperative, intraoperative, and postoperative variables). Multicollinearity was tested using variance inflation factor and proportionality assumptions were evaluated using Schoenfeld residuals. To account for misspecification of Cox models, if any, we conservatively reported robust standard errors. All hypotheses were tested at 0.05 level of significance and the analyses were performed using Stata SE/16.1 (StatCorp, College Station, TX, USA).

## Results

A total of 63 patients were included in the study (Table 1). The mean age was 55.6+ 14.3 years, with 68.3% male and 85.7% Caucasian. The most common indications for pericardiectomy were CP (in 79.4% of subjects) followed by hemorrhagic pericarditis (9.5%), recurrent pericardial effusion (6.4%), and pyopericardium (4.8%). Preoperatively, most subjects were classified as NYHA Class II (33.3%) and III (42.9%). Dyspnea, fatigue and lower extremity edema were the most commonly reported symptoms. Congestive heart failure was present in 90.5% of patients, and 46.0% had a history of prior cardiac intervention. 33.3% had known chronic kidney disease, 15.8% were dialysis-dependent, and 4.7% had a prior renal transplant. None of the study subjects had a prior history of TB.

**Table 1:** Baseline characteristics of total cohort and by overall mortality

	Total (*n* = 63)	Alive (*n* = 47)	Dead (*n* = 16)	*p*-value
Age, years	55.62 ± 14.27	54.79 ± 14.51	58.06 ± 13.69	0.6525
Body Mass Index	31.23 ± 8.31	32.3 ± 8.46	28.08 ± 7.21	0.0511
Gender				0.961
Male	43 (68.25)	32 (68.09)	11 (68.75)	
Female	20 (31.75)	15 (31.91)	5 (31.25)	
Race				0.681
White	54 (85.71)	41 (87.23)	13 (81.25)	
Non-White	9 (14.29)	6 (12.77)	3 (18.75)	
Risk Factors				
Congestive Heart Failure	57 (90.48)	42 (89.36)	15 (93.75)	> 0.999
Prior Cardiac Intervention	29 (46.03)	18 (38.30)	11 (68.75)	0.035
Prior Radiation Exposure	6 (9.52)	3 (6.38)	3 (18.75)	0.166
Hypertension	50 (79.37)	36 (76.60)	14 (87.50)	0.486
Liver Disease	15 (23.81)	12 (25.53)	3 (18.75)	0.740
COPD^a^	16 (25.40)	11 (23.40)	5 (31.25)	0.533
Pneumonia	15 (23.81)	12 (25.53)	3 (18.75)	0.740
Diabetes	19 (30.16)	14 (29.79)	5 (31.25)	0.912
Dyslipidemia	35 (55.56)	26 (55.32)	9 (56.25)	0.948
CKD^b^ - Dialysis	10 (15.87)	4 (8.51)	6 (37.50)	0.013
CKD^b^ - No Dialysis	11 (17.46)	9 (19.15)	2 (12.50)	0.714
Coronary Artery Disease	21 (33.33)	15 (31.91)	6 (37.50)	0.682
Atrial Fibrillation	19 (30.16)	15 (31.91)	4 (25.00)	0.757
Hypothyroidism	13 (20.63)	11 (23.40)	2 (12.50)	0.486
Prior Kidney Transplant	3 (4.76)	2 (4.26)	1 (6.25)	> 0.999
Autoimmune Disease	8 (12.70)	6 (12.77)	2 (12.50)	> 0.999
Illicit Drug Use	8 (12.70)	7 (14.89)	1 (6.25)	0.667
Immunocompromised	10 (15.87)	5 (10.64)	5 (31.25)	0.051
Pericardial Effusion	34 (53.97)	24 (51.06)	10 (62.50)	0.428
Prior Pericardiocentesis	11 (17.46)	8 (17.02)	3 (18.75)	> 0.999
Prior Pericardial Window	11 (17.46)	7 (14.89)	4 (25.00)	0.448
Pleural Effusion	23 (36.51)	14 (29.79)	9 (56.25)	0.058
Redo Sternotomy	9 (14.29)	4 (8.51)	5 (31.25)	0.039
LVEF^c^	54.48 ± 10.70	54.53 ± 10.06	54.31 ± 12.97	0.8792
Reason for Surgery				0.217
Constrictive Pericarditis	50 (79.37)	36 (76.60)	14 (87.50)	
Hemorrhagic Pericarditis	6 (9.52)	6 (12.77)	0	
Recurrent Pericardial Effusion	4 (6.35)	2 (4.26)	2 (12.50)	
Pyopericardium	3 (4.76)	3 (6.38)	0	
NYHA^d^ Class on Admission				0.386
I	6 (9.52)	6 (12.77)	0	
II	21 (33.33)	16 (34.04)	5 (33.33)	
III	27 (42.86)	20 (42.55)	7 (46.67)	
IV	7 (11.11)	4 (8.51)	3 (20.00)	
Not Available	2 (3.17)	1 (2.13)	1 (6.25)	
Presenting Symptoms				
Fever	5 (7.94)	4 (8.51)	1 (6.25)	> 0.999
Nausea	5 (7.94)	5 (10.64)	0	0.317
Dyspnea	57 (90.48)	41 (87.23)	16 (100.00)	0.324
Fatigue	55 (87.30)	40 (85.11)	15 (93.75)	0.667
Lower Extremity Edema	36 (57.14)	26 (55.32)	10 (62.50)	0.616
Chest Pain	31 (49.21)	23 (48.94)	8 (50.00)	0.941
Abdominal Distension	30 (47.62)	22 (46.81)	8 (50.00)	0.825
Creatinine, mg/dL	1.09 (0.66)	1.01 (0.61)	1.5 (1.21)	0.0067
Medical Management				
Diuretics	41 (65.08)	32 (68.09)	9 (56.25)	0.391
NSAID^e^	23 (36.51)	19 (40.43)	4 (25.00)	0.371
Colchicine	5 (7.94)	5 (10.64)	0	0.317
**Intraop Variables**				
Procedure Performed				0.222
Isolated Pericardiectomy	42 (66.67)	29 (61.70)	13 (81.25)	
Pericardectomy + Concomitant Procedure	21 (33.33)	18 (38.30)	3 (18.75)	
CPB^f^ utilization	23 (36.51)	20 (42.55)	3 (18.75)	0.133
CPB time, minutes	162 ± 89.96	153.28 ± 88.99	214.33 ± 93.93	0.247
Cross Clamp time, minutes	110.43 ± 82.55	107.5 ± 81.70	128 ± 120.21	> 0.999
Status				0.038
Elective	41 (65.08)	34 (72.34)	7 (43.75)	
Urgent	22 (34.92)	13 (27.66)	9 (56.25)	

Values are expressed as n(%) and mean + standard deviation unless otherwise noted

^a^chronic obstructive pulmonary disease

^b^chronic kidney disease

^c^left ventricular ejection fraction

^d^New York Heart Association

^e^non-steroidal anti-inflammatory drug

^f^cardiopulmonary bypass

All patients had been previously been treated medically, with 67.4% on diuretics, 26.5% on nonsteroidal anti-inflammatories (NSAIDs), and 4.1% on colchicine in the immediate period leading up to surgery. 34.9% had undergone prior pericardiocentesis or pericardial window to address symptoms. Mean left ventricular ejection fraction (LVEF) was 54.5% + 10.7%. Erythrocyte sedimentation rate (ESR) and C-reactive protein (CRP) values were available only in seven subjects, but of these patients, all had elevation of the inflammatory markers. The majority of patients underwent multiple diagnostic studies to confirm hemodynamically significant pericarditis: 61.2% had both computed tomography (CT) and cardiac catheterization, while 18.4% underwent CT, cardiac catheterization, and magnetic resonance imaging (MRI). Mean duration between diagnosis and surgery was 77.8 days.

65.1% of cases were elective status, with the remainder classified as urgent due to other concomitant cardiac pathology. Isolated pericardiectomy was performed in 66.7% of cases. In instances where additional cardiac surgical procedures were performed, the most common were coronary artery bypass grafting (CABG), aortic valve replacement (AVR), and aortic aneurysm repair at 14.3, 11.1 and 6.4%, respectively. Of all pericardiectomy procedures, 96.8% were subtotal or anterior pericardiectomy (extent of resection from phrenic nerve to phrenic nerve and from superior vena cava to inferior vena cava) while 3.2% were total pericardiectomy (resection of both anterior and posterior pericardium, leaving bilateral pedicled phrenic nerves). Cardiopulmonary bypass (CPB) was utilized in 36.5% of cases with a mean CPB time of 162.0 + 90.0 min. Aortic crossclamp was performed in 27.0% of cases with a mean crossclamp time of 110.4 + 82.5 min.

Mean postoperative LOS was 15 days (Table 2). The most common postoperative adverse events were pneumonia (11.1%), sternal infection (9.5%), and sepsis (7.9%). 30-day readmission rate was 25.4%, with the most frequent causes for readmission being congestive heart failure (37.5%) and chest pain (12.5%). Pathology specimen results were available in 77.8% of cases, and the most common findings were fibrosis (71.4%), chronic inflammation (38.8%), calcification (22.4%), and organizing thrombus (20.4%). The mean follow-up interval was 1132 days. At first post-discharge follow-up, the majority of subjects were NYHA Class I (57.1%) and II (14.3%). Over the entire follow-up period, three patients (4.8%) had symptomatic recurrence: two received only medical management while one underwent re-do pericardiectomy 645 days after initial surgery.

**Table 2:** Postoperative outcomes

	Total (*n* = 63)	Alive (*n* = 47)	Dead (*n* = 16)	*p*-value
Mortality				
1-year	6 (9.52)	–	–	
3-year	9 (14.29)	–	–	
5-year	13 (20.63)	–	–	
Overall (10-year)	16 (25.40)			
LOS^a^, days	15.38 ± 16.68	13.38 ± 16.71	21.25 ± 15.63	0.0213
Postoperative Ventilation, hours	33.19 ± 71.93	27.67 ± 64.43	49.06 ± 90.69	0.7463
Reintubation	6 (9.52)	2 (4.26)	4 (25.00)	0.032
Surgical Site Infection	6 (9.52)	4 (8.51)	2 (12.50)	0.639
Sepsis	5 (7.94)	2 (4.26)	3 (18.75)	0.099
Bleeding	2 (3.17)	1 (2.13)	1 (6.25)	0.446
Pneumonia	7 (11.11)	3 (6.38)	4 (25.00)	0.063
Renal Failure	3 (5.66)	2 (4.65)	1 (10.00)	0.473
Readmission ≤30 Days	16 (25.40)	12 (25.53)	4 (25.00)	> 0.999
Recurrent Pericarditis	3 (4.76)	3 (6.38)	0	0.564
NYHA^b^ Class at First Encounter after Discharge				0.244
I	36 (57.14)	27 (57.45)	9 (56.25)	
II	9 (14.29)	8 (17.02)	1 (6.25)	
III	6 (9.52)	3 (6.38)	3 (18.75)	
IV	5 (7.94)	5 (10.64)	0	
Not Available	7 (11.11)	4 (8.51)	3 (18.75)	

Values are expressed as n(%) or mean + standard deviation unless otherwise noted

^a^length of stay

^b^New York Heart Association

**Fig. 1:**
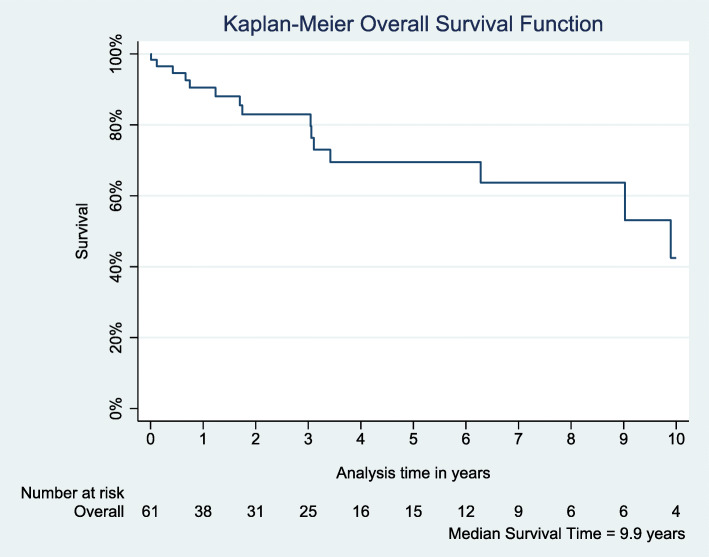
Survival curve for overall cohort

There were no intraoperative or 30-day deaths. Mortality at 1, 3, 5, and 10 years was 9.5, 14.3, 20.6, and 25.4% respectively (Fig. 1). Bivariate analysis indicated that prior cardiac intervention (*p* = 0.035), dialysis (*p* = 0.013), re-do operation (*p* = 0.039), elevated creatinine (*p* = 0.007), surgery status (*p* = 0.038), postoperative LOS (*p* = 0.021), and reintubation (*p* = 0.032) were significant risk factors associated with mortality (Tables 1 and 2). Multivariable Cox Proportional Hazard Ratio (HR) analysis revealed that prior radiation exposure (HR 2.57, *p* = 0.044), dialysis (HR 22.06, *p* < 0.0001), and postoperative reintubation (HR 46.35, *p* < 0.0001) are associated with mortality risk (Table 3).

**Table 3:** Multivariable Cox proportional hazard analysis for isolated pericardiectomy

	Overall Survival**	
	Hazard Ratio(95%CI)	*p*-value
Immunocompromised		
No	**REF**	
Yes	2.94 (0.67–12.80)	0.151
Radiation Exposure		
No	**REF**	
Yes	2.57 (1.03–6.46)	0.044
CKD^a^-Dialysis		
No	**REF**	
Yes	22.06 (7.51–64.83)	< 0.0001
LOS^b^	1.04 (1.00–1.10)	0.131
Postoperative Reintubation		
No	**REF**	
Yes	46.35 (8.81–243.97)	< 0.0001
Postoperative Pneumonia		
No	**REF**	
Yes	0.01 (0.00–0.13)	< 0.0001

** Cox Regression

^a^chronic kidney disease

^b^length of stay

## Discussion

Pericarditis remains an infrequent cause of hospital admissions requiring definitive surgical pericardiectomy, even at high volume cardiac centers. Given the relatively variable and high mortality rates following pericardiectomy, there has been significant interest in studying outcomes and risk factors [1–3, 7, 8, 14, 21–23]. Our short- and long- term results are similar to those presented by other investigators [1, 8, 21]. However, some published reports indicated a significantly higher perioperative mortality; this may be due to differences in baseline patient characteristics as such studies often have a higher proportion of subjects classified as preoperative NYHA Class IV [2, 5–8, 11, 14, 24]. We find that there are a number of risk factors that affect survival after pericardiectomy, with the most significant being the presence of prior cardiac intervention, baseline chronic kidney disease with dialysis, and prior radiation exposure. This is not surprising, as all are known risk factors for adverse outcomes following other cardiac surgical procedures. Mediastinal radiation often leads to fibrosis of mediastinal contents, including the pericardium, which subsequently can lead to constrictive physiology [20]. Similar to other published reports, the overall mortality rate for our study subjects with a history of mediastinal radiation was 50% at 10 years, which is about three times greater than those without prior mediastinal radiation [2, 3, 5, 6, 14]. Uremia secondary to renal failure is a known risk factor for development of CP and also for mortality following cardiac surgery. Outcomes after pericardiectomy are no exception: of the 16 patients who died during the 10-year follow-up interval, six (37.5%) had chronic kidney disease or were dialysis-dependent.

Preoperative LVEF was not associated with increased postoperative mortality. The majority of study subjects had normal LVEF but with reported symptoms of heart failure including dyspnea, fatigue, and extremity edema. This is explained by the physiology of CP, which is primarily a diastolic pathology with restricted cardiac filling rather than impaired ventricular contractility and ejection. There was marked improvement in symptoms postoperatively, with most patients improving to NYHA Class I and II following surgery. This finding is again similar to that reported in the literature, providing further evidence of the utility of pericardiectomy in improving quality of life for these patients [1, 6, 8, 23–26].

Up to 90% cases of pericarditis in the United States and Europe occur after a viral infection with patients recalling an episode of flu-like illness or gastroenteritis before the onset of pericarditis symptoms [9, 10, 13]. Apart from direct pericardial infection, viral syndromes can also trigger an autoimmune reaction due to molecular mimicry and can lead to the formation of superantigens [9]. In our study only 11.1% of the patients reported a history of viral illness. We suspect this is likely attributable to the subjective nature of this particular aspect of medical history; patients may not have specifically been asked at the time of treatment about a history of viral illness, and in those cases, patients may have had a tendency to not consider past viral symptoms relevant or significant enough to report.

While pericardiectomy can also be performed via a thoracotomy or subxiphoid approach, all patients in our cohort underwent median sternotomy [8]. We continue to favor this approach as it provides the greatest exposure and options to utilize CPB if necessary. The majority of the isolated pericardiectomy operations in the study were performed without CPB. The benefits to this approach include lower risk of bleeding and avoidance of aortic cannulation and its attendant risks. However, pericardiectomy without CPB support might result in suboptimal resection: in our study, all three patients who suffered recurrent pericarditis during the follow-up period had undergone pericardiectomy without the use of CPB. This association was not statistically significant but may indicate a topic worthy of future study. Extent of pericardial resection is also debated, with published data that seem to support either subtotal or total pericardiectomy without clear definitive superiority of one over the other [1, 2, 8, 23, 27, 28]. In our study, we did not find a notable relationship between extent of resection and recurrence of symptoms. Mitigation of recurrence risk is a realm of ongoing study; novel techniques such as the use of allograft stem cells or amniotic membrane patch during pericardiectomy may show promise in this regard [29].

Limitations of this study are related to its retrospective nature and the relatively small sample size. Follow-up was based on medical documentation rather than direct patient communication, and we were unable to capture those subjects that were lost to follow-up and/or sought subsequent medical care at an outside institution. Exact cause of death could not be ascertained unless patients died within our hospital system. Furthermore, the operating surgeons between the early study period and late study period were completely different, which may contribute to differences in outcomes. Lastly, this study is unable to capture outcomes for pericardiectomy performed for TB-induced pericarditis, which is the most common cause outside the United States. Studies conducted in India and Nepal, for instance, reported the vast majority of patients undergoing pericardiectomy had a history of TB or had received medical antibiotics treatment for TB prior to surgery [17, 24, 25, 30, 31].

## Conclusion

Although relatively rare, pericardiectomy remains the only definitive treatment option for pericarditis that is refractory to medical management. Successful pericardiectomy can lead to symptomatic and NYHA Class improvement but carries moderate to high intermediate- and long- term mortality risk particularly in those with significant underlying medical co-morbidities. Areas of future study can include methods to reduce the incidence of constrictive pericarditis as well as techniques to reduce the risk of recurrence following pericardiectomy.

## Data Availability

The datasets analyzed in the current study are available from the corresponding author on reasonable request.
